# Indocyanine Green Near-Infrared Fluorescence Imaging-Guided Laparoscopic Heminephrectomy for Left Ureteral Cancer in Patient with Horseshoe Kidney

**DOI:** 10.1155/2019/4859301

**Published:** 2019-05-02

**Authors:** Takao Natsuyama, Yozo Mitsui, Masato Uetani, Shigeyuki Ohta, Shin-ichi Hisasue

**Affiliations:** ^1^Department of Urology, Chiba-Nishi General Hospital, 270-2251 Chiba, Japan; ^2^Department of Urology, Toho University Faculty of Medicine, 143-8540 Tokyo, Japan

## Abstract

Laparoscopic surgery for patients with a horseshoe kidney is challenging because of the location, aberrant vasculature, and difficulty with division of the isthmus with adequate hemostasis. We herein report performance of a laparoscopic heminephrectomy for left ureteral cancer in a patient with a horseshoe kidney under guidance from near-infrared fluorescence (NIRF) imaging using indocyanine green (ICG). A 62-year-old male was referred to our hospital for treatment of left ureteral cancer associated with a horseshoe kidney. We performed a laparoscopic left nephroureterectomy and bladder resection in June 2017. During the operation, the NIRF imaging system was used to evaluate the border of the kidney parenchyma isthmus after ligation of the left kidney vasculature supply. Interestingly, the dominant region of the right kidney showed strong ICG fluorescence as compared to the left kidney region. With the assistance of ICG-based NIRF imaging, isthmus division was performed with monopolar scissors and adequate hemostasis was obtained by electrocautery coagulation. This is the first report of use of an ICG-based NIRF imaging system and this novel approach can help to demarcate the left moiety isthmus from right one with more certainty.

## 1. Introduction

A horseshoe kidney, one of the most common renal fusion anomalies, is associated with various urological diseases, such as urolithiasis, ureteropelvic junction obstruction, and urinary infection, though rarely malignant, which may require a surgical procedure for treatment. A laparoscopic approach in a case with a horseshoe kidney is challenging because of location, as well as vascular anomalies and the kidney parenchyma isthmus that connects the two sides. Good preoperative understanding of the anatomical structure of a horseshoe kidney based on radiological imaging as well as functional intraoperative visualization may contribute to more safe and reliable laparoscopic surgery.

Near-infrared fluorescence (NIRF) imaging with indocyanine green (ICG) is a novel intraoperative technique that can be applied in a variety of clinical settings, such as for confirmation of blood flow and detection of sentinel lymph nodes, as well as cancer detection. We previously reported that normal kidney tissues showed high fluorescence with ICG administration [1, 2], which led us to speculate that this novel technology could be applied for intraoperative discrimination of the isthmus of a horseshoe kidney. We herein report a case of left ureteral cancer in a patient with a horseshoe kidney who was successfully treated for the first time by a laparoscopic heminephrectomy under the aid of intraoperative ICG-based NIRF imaging.

## 2. Case Presentation

**Figure 1:**
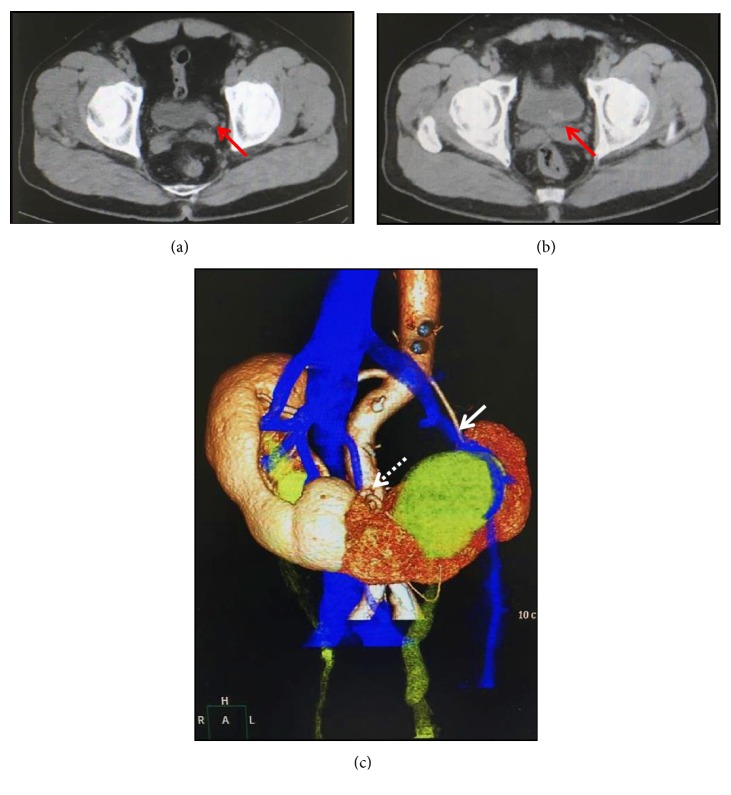
(a), (b) Pelvic CT images showing a left lower ureteral tumor near the ureteral orifice (red arrows). (c) Three-D CT imaging. The left renal artery (white arrow) and an aberrant renal artery (white dotted arrow) supplied the left part of the horseshoe kidney.

A 62-year-old Japanese male with a horseshoe kidney was referred to our hospital for further examination of left hydronephrosis. The patient was obese with a body mass index of 32.0 kg/m^2^ (height 170 cm, body weight 92 kg) and had undergone medical treatment for hypertension. Laboratory examination results showed mild renal dysfunction with a serum creatinine level of 1.21 mg/dL and estimate glomerular filtration rate of 48.2 mL/minute. Computed tomography (CT) revealed a left lower ureteral mass near the ureteral orifice, resulting in renal hydronephrosis (Figures 1(a) and 1(b)). Furthermore, three-dimensional (3-D) CT imaging showed a normal renal vascular system, while an aberrant renal artery located just below the root of the inferior mesenteric artery (IMA) was found to supply the bilateral sides of the lower part of the horseshoe kidney (Figure 1(c)). In addition, urine cytology findings were positive for malignancy. Based on these findings, we diagnosed a left lower ureteral tumor associated with a horseshoe kidney.

**Figure 2:**
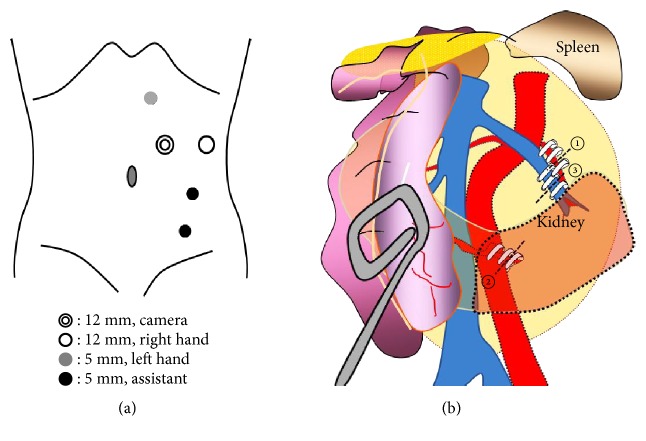
(a) Laparoscopic ports used in this case. (b) Schema of procedure for ligation of the left renal vascular system. The main renal artery and an aberrant renal artery were ligated and divided in that order; then the renal vein was cut.

**Figure 3:**
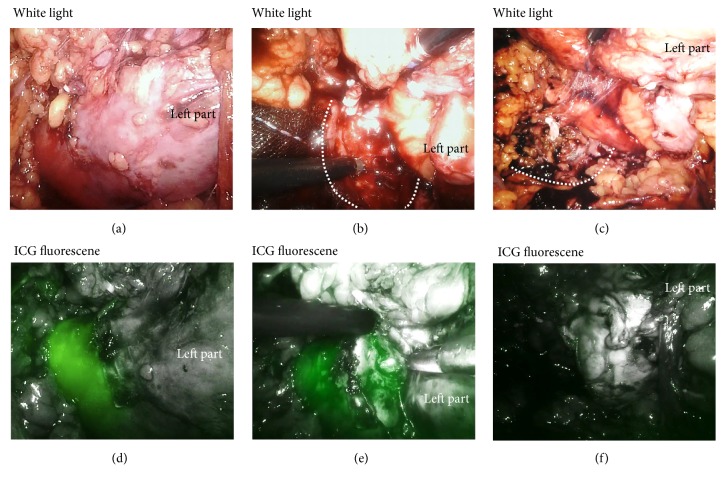
(a) Gross appearance of horseshoe kidney under white light after blockage of blood supply to the left kidney. (b) Resection of the renal isthmus under white light. Dotted circle indicates renal isthmus border. (c) Resected surface of right part of kidney under white light. Adequate hemostasis was obtained by bipolar coagulation and a fibrin patch. Dotted circle indicates renal isthmus border. (d) ICG-based NIRF images showing strong ICG fluorescence in the dominant region of the right part of the kidney after blockage of blood supply to the left kidney. (e) Proper resection of the renal isthmus could be performed with assistance from NIRF imaging. (f) Adequate hemostasis of the resected surface of the right part of the kidney was confirmed using ICG-based NIRF imaging.

In June 2017, laparoscopic left nephroureterectomy and bladder resection procedures were performed with 5 laparoscopic ports, with the port schema shown in Figure 2(a). Using an intraperitoneal approach, we cut the peritoneum and dislocated the intestine to approach the renal helium. After visualizing the left renal vascular system, the main renal artery supplying the left side of the upper part of the horseshoe kidney and left branch of the aberrant renal artery passing under the IMA were ligated by use of a Hem-o-lok® polymer clip and divided, after which the left renal vein was ligated and divided in the same manner (Figure 2(b)). Next, we were able to observe the renal isthmus using an NIRF system (da Vinci Xi FireFlyTM®) at 1 minute after intravenous administration of 1 mL of ICG (Diagnogreen 0.25%; Daiichi Pharmaceutical, Tokyo, Japan). Strong ICG fluorescence was observed in the dominant region of the right part of the kidney, while no fluorescence was noted in the left kidney region (Figure 3(d)). These findings indicated that blood supply to the left side of the kidney was completely blocked, which was more apparent as compared to white light images (Figure 3(a)). Subsequently, the heminephrectomy was started under the assistance of NIRF imaging using cold scissors. During excision, visualization with white light and the NIRF was switched at the discretion of the operator in order to confirm the plane of excision between the right and left parts of the kidney (Figures 3(b) and 3(e)). Adequate hemostasis from the resected area could be obtained by electrocautery bipolar coagulation and use of a TacoSil® tissue sealing sheet, with no suturing required (Figures 3(c) and 3(f)). Following completion of the laparoscopic heminephrectomy, the patient was placed in a supine position and partial resection of the bladder was performed. The total operation time was 5 hours 21 minutes and blood loss was 410 mL (obtained in urine from the bladder). A histopathological examination revealed an invasive urothelial carcinoma (grade 2>1, INF*β*, pT2a, ly0, v0, u-lt0, RM0). There were no perioperative complications and the patient was discharged 9 days after surgery.

## 3. Discussion

A horseshoe kidney is one of the most commonly encountered kidney fusion anomalies, with an incidence rate estimated to be 1 in 400 in the general population. This condition is often accompanied by other urologic disorders and a surgical procedure such as a heminephrectomy may be necessary in some cases. Recently, minimally invasive surgical techniques including a laparoscopic approach have been widely applied for urological surgery, a trend that also applies to diseases associated with a horseshoe kidney. For example, Tuncel et al. reported the efficacy and safety of a laparoscopic heminephrectomy in a case of horseshoe kidney [3]. To enhance the utility and safety of such an approach, we used an ICG-based NIRF imaging system during performance of a laparoscopic heminephrectomy for left ureteral cancer associated with a horseshoe kidney. Our results are the first to show that this system may be a promising intraoperative imaging technique to facilitate laparoscopic attempts for performance of a heminephrectomy.

NIRF imaging technology, which can be utilized in real time during surgery, has been applied for a variety of surgical specialties over the past decade. Several fluorescent tracers can be used, of which ICG is most widely selected because it has few side effects and its pharmacokinetics are well understood. Following an intravenous injection, most of the ICG binds to serum protein, which allows fluorescence to be visualized throughout the vascular system, while in well-perfused normal renal parenchyma, fluorescence can be observed from approximately 1 minute after ICG administration [1, 2]. In the present case, the dominant region of the right kidney showed strong green fluorescence with NIRF imaging, while no fluorescence was seen in the ischemic region of the left kidney, suggesting that all renal arteries in the left side were completely blocked. Preoperative 3D CT images are very useful to identify horseshoe kidney vessels [4–6], though the complicated vessel anatomy associated with this condition can sometimes lead to overlooked small aberrant vessels. We consider that use of an ICG-based NIRF imaging system complements preoperative radiological imaging by providing real-time intraoperative renal angiogram images.

A successful heminephrectomy requires not entering the renal collecting system of the contralateral kidney and achievement of adequate bleeding control [3]. When dividing the isthmus, we repeatedly referred to images from the ICG-based NIRF system, as they assisted in determining the proper direction of the incision. As shown in Figure 3(e), this system enabled intraoperative discrimination between the ischemic and well-perfused renal parenchyma and helped to avoid entering the right kidney renal collecting system. Also, sufficient hemostasis was obtained by use of bipolar coagulation and a fibrin patch, indicating that proper resection of the isthmus with assistance from ICG-based NIRF imaging contributed to reducing the amount of bleeding in our case. In addition, the technique was not difficult and did not affect the time needed for division of the isthmus. Based on our findings, we concluded that the ICG-based NIRF imaging system enhances the safety and reliability of a laparoscopic heminephrectomy procedure.

The urological surgery procedure that most often utilizes the ICG-based NIRF imaging system is robotic-assisted partial nephrectomy for renal tumors [7]. Under guidance provided by the system, a renal tumor appears hypofluorescent as compared to the normal kidney parenchyma because of an absence in the tumor of bilitranslocase, a carrier protein of ICG present in normal proximal tubule cells [8], allowing for clear discrimination between cancer and normal kidney tissues. In general, renal tumors associated with a horseshoe kidney are more rare than upper urinary tract urothelial tumors, though approximately 200 cases have been described thus far and robotic approaches have recently been attempted [6, 9, 10]. We speculate that the ICG-based NIRF imaging system can also help with minimally invasive surgery for renal tumors associated with a horseshoe kidney.

In conclusion, a laparoscopic heminephrectomy for left ureteral cancer in a patient with a horseshoe kidney was performed safely and reliably with guidance from ICG-based NIRF imaging. This novel system may play an important role in minimally invasive surgery for various horseshoe kidney-associated diseases.
